# DIABETES, SELF-EFFICACY TOWARD DIABETES PREVENTIVE BEHAVIORS, AND DEPRESSIVE SYMPTOMS AMONG KOREAN AMERICANS

**DOI:** 10.1093/geroni/igz038.2612

**Published:** 2019-11-08

**Authors:** Soyeon Cho

**Affiliations:** City University of New York-New York City College of Technology, Brooklyn, New York, United States

## Abstract

Type 2 diabetes is a largely preventive chronic disease, which requires persevering self-management by maintaining healthy life style. Prevalence of Type 2 diabetes among Asian Americans are rapidly increasing, yet little is known about Asian Americans’ self-efficacy towards diabetes preventive behaviors. Thus, the present study examined self-efficacy on diabetes preventive behaviors (DPB) as a potential mediator in the association between diabetes and depressive symptoms among older Korean Americans. Data were driven from a cross-sectional study of 235 community-dwelling Korean American older adults (aged 60 and older) in 2013. The direct significant relation between diabetes and depressive symptoms became insignificant after self-efficacy on DPB was introduced, which demonstrates a full mediation effect of self-efficacy on DPB. Results suggest that even in the presence of diabetes, mental well-being such as depression of older adults can be maintained by having competence in self-management of their own health.

